# Optimizing Treatment of Antimicrobial-resistant *Neisseria gonorrhoeae*

**DOI:** 10.3201/eid1108.050157

**Published:** 2005-08

**Authors:** Kakoli Roy, Susan A. Wang, Martin I. Meltzer

**Affiliations:** * Centers for Disease Control and Prevention, Atlanta, Georgia, USA

**Keywords:** Neisseria gonorrhoeae, Antimicrobial resistance, Cost-effectiveness analysis

## Abstract

The increasing prevalence of ciprofloxacin-resistant *Neisseria gonorrhoeae* has required replacing inexpensive oral ciprofloxacin treatment with more expensive injectable ceftriaxone. Further, monitoring antimicrobial resistance requires culture testing, but nonculture gonorrhea tests are rapidly replacing culture. Since the strategies were similar in effectiveness (>99%), we evaluated, from the healthcare system perspective, cost-minimizing strategies for both diagnosis (culture followed by antimicrobial susceptibility tests versus nonculture-based tests) and treatment (ciprofloxacin versus ceftriaxone) of gonorrhea in women. Our results indicate that switching from ciprofloxacin to ceftriaxone is cost-minimizing (i.e., optimal) when the prevalence of gonorrhea is >3% and prevalence of ciprofloxacin resistance is >5%. Similarly, culture-based testing and susceptibility surveillance are optimal when the prevalence of gonorrhea is <13%; nonculture-based testing is optimal (cost-minimizing) when gonorrhea prevalence is ≥13%.

## Abstract 2

Optimal point to switch to more expensive drug depends on prevalence of drug resistance and

disease in the population.

Gonorrhea is the second most frequently reported sexually transmitted disease (STD) in the United States (1,2) infecting an estimated 800,000 people at a cost of $1 billion annually (3,4). In women, untreated gonorrhea can lead to pelvic inflammatory disease (PID) and to profound long-term sequelae such as chronic pelvic pain, ectopic pregnancy, and infertility (5). In addition, gonococcal infections have been shown to facilitate transmission of HIV (6). Gonorrhea is frequently asymptomatic in women, which creates a large pool of undetected infections. These infections go untreated, which may increase the probability of disease progression in the patient and transmission to sexual partners.

An important obstacle in the control of gonorrhea is the emergence of antimicrobial–resistant strains. As ciprofloxacin-resistant *Neisseria gonorrhoeae* strains become more prevalent in the United States (7–10), treatment with cephalosporins such as cefixime and ceftriaxone becomes necessary (10,11). Antimicrobial susceptibility testing requires that *N. gonorrhoeae* be grown in culture. However, the availability of affordable and accurate nonculture tests, along with the convenience of combination nonculture tests for *N. gonorrhoeae* and *Chlamydia trachomatis*, has resulted in the increased use of nonculture-based tests^1^ (12). The resulting reduction in the use of culture-based testing poses challenges for monitoring antimicrobial resistance.

To the best of our knowledge, the economic consequences of diagnostic test and treatment selection in the face of rising antimicrobial resistance for *N. gonorrhoeae* have not been explored in the literature. Our objective was to identify the most cost-effective combination of diagnostic test (culture or nonculture) and treatment (ciprofloxacin or ceftriaxone) for gonorrhea when the incidence of ciprofloxacin-resistant *N. gonorrhoeae* infections is increasing.

## Methods

### The 4 Strategies

We evaluated and compared the cost and disease outcomes associated with 4 strategies, identified from current practice and consultations with experts, for diagnosing and treating gonorrhea in women (Table 1). The 2 treatments evaluated were a single, oral 500-mg dose of ciprofloxacin (for which gonococcal resistance exists in some parts of the United States), and a single, 125-mg dose of ceftriaxone by intramuscular injection (for which we assumed no resistance has been identified). Two of the strategies used culture-based tests followed by antimicrobial susceptibility testing, while the remaining 2 strategies used nonculture-based combination tests, such as nucleic acid amplification assay or the nucleic acid hybridization test, for detecting both *N. gonorrhoeae* and *C. trachomatis*. Since we know that resistance to ciprofloxacin already exists, we assume that 80% of culture-positive specimens would also be tested for antimicrobial susceptibility when ciprofloxacin was used for treatment (ST1). Ideally, 100% of culture-positive specimens would be tested for antimicrobial susceptibility, but we allow for some losses because of incomplete tracking, handling, and transportation. In the absence of recorded treatment failure caused by antimicrobial resistance to ceftriaxone, we assume that 20% of specimens would be susceptibility tested for surveillance purposes.

**Table 1:** Strategies modeled

Strategy (ST)	Brief description	Detailed description
ST1	Ciprofloxacin + culture tests + ciprofloxacin susceptibility tests	Prescribe ciprofloxacin to symptomatic patients and culture test all patients. Test 80% of all positive specimens for ciprofloxacin resistance. Recall and treat asymptomatic gonorrhea patients and patients with ciprofloxacin-resistant strains.
ST2*	Ciprofloxacin + nonculture tests	Prescribe ciprofloxacin to symptomatic patients and use nonculture tests on all patients. Recall and treat positive asymptomatic gonorrhea patients.
ST3	Ceftriaxone + culture tests + ceftriaxone susceptibility tests	Prescribe ceftriaxone to symptomatic patients and culture test all patients. Recall and treat asymptomatic gonorrhea patients. Test 20% of positive isolates for resistance to cephalosporin.
ST4*	Ceftriaxone + nonculture tests	Prescribe ceftriaxone to symptomatic patients and use nonculture tests on all patients. Recall and treat asymptomatic gonorrhea patients.

*Since ST2 and ST4 do not use culture-based testing, no antimicrobial susceptibility tests are assumed to be associated with these strategies.

For all 4 strategies, we assume that women with symptoms of gonorrhea who go to a healthcare provider will be presumptively treated with the antimicrobial agent indicated by the strategy. In all strategies, all women undergo testing for gonorrhea with either culture- or nonculture-based tests. All strategies assume that attempts will be made to recall and treat asymptomatic women who test positive. Two strategies (ST1 and ST3, Table 1) further assume that those symptomatic women found to be infected by a resistant strain will be recalled and retreated. Successful treatment of the detected patients depends on the effectiveness of the antimicrobial therapy, given the particular susceptibility patterns of gonococcal strains in a geographic location.

### Economic Evaluation Methods

To conduct an economic evaluation of the 4 strategies from a healthcare system perspective, we built a decision tree by using DATA 4.0 (TreeAge Software Inc., Williamstown, MA, USA). Appendix 1 contains schematics of the tree. The gonorrhea-related health outcomes included in the evaluation of each strategy were PID and PID-associated sequelae (chronic pelvic pain, ectopic pregnancy, and infertility). We also included the probability and associated costs of female-to-male transmission of gonorrhea (assuming that all couples are heterosexual). If the male partner is infected, either urethritis or epididymitis could develop and the infection could be transmitted to another female partner (or the original female partner could be reinfected after she has been cured of the initial infection). In the initial female-to-male transmission of gonorrhea, the additional probability and associated costs of cotransmission of HIV exist. Economic outcomes include all diagnostic test-related costs (i.e., cost of supplies, equipment, and labor), direct medical costs of treatment (for pelvic examination and patient recall), cost of treating PID and sequelae (i.e., inpatient and outpatient medical costs), and costs of transmission of gonorrhea and HIV to sex partners (assuming that all couples are heterosexual).

For each strategy (Table 1), we used the decision tree to calculate the expected cost per case of gonorrhea treated, the expected proportion of cases successfully treated (case-patients with no PID or sequelae), and expected cost per case-patient successfully treated. Average and incremental cost-effectiveness analyses, also conducted for a hypothetical cohort of 1 million women treated with each of the 4 alternative strategies, are contained in Appendix 2.

## Data

### Probabilities

The probabilities used were derived from a review of the published literature, expert opinion, and unpublished data from local, state, and national sources (Table 2). In the base case, the prevalence of gonococcal infection among women was assumed to be 0.5% (range 0%–15%). We assumed that of all gonorrhea-infected women who enter the clinic, 30% (range 20%–60%) display urogenital symptoms for gonorrhea, and 70% do not display such symptoms. We also assume among women who enter the clinic, but are uninfected with gonorrhea, that 20% (range 0%–40%) will be presumptively treated for gonorrhea due to nonspecific urogenital symptoms associated with both gonorrhea and other STDs (13–15). All women with untreated cases of gonorrhea have a 16% probability of developing PID (15,16).

**Table 2:** Input probabilities

Variable description	Probabilities (%)			
	Base	Range	Distribution*	Sources
Prevalence of gonorrhea in community among women	1.0	0–15	Triangular	2
Prevalence of ciprofloxacin-resistant *Neisseria gonorrhoeae*	0.1	0–20	Triangular	7
Prevalence of ceftriaxone-resistant *N. gonorrhoeae*	0			Assumed
Treatment failure when strain is resistant to antimicrobial agent	100			Assumed†
Treatment failure when strain is not resistant to antimicrobial agent	0			Assumed†
Infected with gonorrhea and symptomatic	30	20–50	Triangular	5,13,14
Infected with gonorrhea but without symptoms‡	70	Residual‡		Calculated
Not infected but with gonorrhea symptoms	20	10–40	Triangular	5,13,15
Not infected and without gonorrhea symptoms‡	80	Residual‡		Calculated
Recalled patient returning to clinic	40	20–80	Triangular	16,17
Sensitivity of nonculture-based tests	95	85–100	Triangular	14,18,19
Specificity of nonculture-based tests	97	95–99	Triangular	14,18,19
Sensitivity of culture-based tests	93	85–95	Triangular	14,18,19
Specificity of culture-based tests	97	95–97	Triangular	14,18,19
Concurrent HIV transmission§	0.066	0–0.5	Triangular	20
Develop pelvic inflammatory disease (PID) and sequelae, among untreated gonorrhea cases	16	10–40	Triangular	5,13,14,21
Development of PID only (no sequelae)¶	70	70–72	Uniform	15,16,21,22
Developing sequelae of PID¶				
Infertility	6	1–6	Uniform	15,16,21
Ectopic pregnancy	8	5–9	Uniform	15,16,21
Chronic pelvic pain	16	15–20	Uniform	15,16,21
Urethritis	50	35–65	Uniform	15,16,21
Epididymitis	2	1–5	Uniform	15,16,21
For strategy 1, % of culture-positive samples tested for antimicrobial susceptibility#	80			Assumed
For strategy 3, % of culture-positive samples tested for antimicrobial resistance#	20			Assumed
Female-to-male transmission of gonorrhea§	50	30–75	Uniform	5,13,23
Male-to-female transmission of gonorrhea§	50	30–75	Uniform	5,13,23

*The probability distributions used in the Monte Carlo sensitivity analysis. Uniform distributions were constructed with the minimum and maximum of the given ranges. Triangular distributions were constructed with the minimum and maximum of the given ranges and the base case as the "most likely" value. †Assumes 0% drug failure if organism is not resistant. ‡The probability of being infected and without gonorrhea symptoms is the residual value after considering the probability of being infected and with gonorrhea symptoms. Likewise, the probability of being not infected and without gonorrhea symptoms is the residual value after considering the probability of not being infected and with gonorrhea symptoms. §Describes probability of initially infected woman transmitting disease to male partner, who then has the probability of infecting another female partner (or reinfecting original female partner after she has been cured of initial infection). Further, with the initial female-to-male transmission, the probability of concurrent HIV transmission exists. ¶Rate of PID (only) and rates of PID-related sequelae are given as percentages of those that develop PID. #See Table 1 for descriptions of strategy 1 (ST1) and strategy 3 (ST3). In ST1, culture-positive samples are tested for ciprofloxacin resistance. In ST3, culture-positive samples are tested for ceftriaxone resistance.

Previous studies used an estimate that 80% of women notified of a positive test result returned for treatment (16,17). However, in the absence of additional supportive data, we assumed recall rates, for both asymptomatic patients and those infected with a resistant strain, to be 40%. To simplify the model, we further assumed that infection with a resistant strain would lead to complete treatment failure. In reality, antimicrobial resistance is often not absolute, and successful treatment may still occur when a patient is infected with a resistant strain. This assumption biases the results toward switching from ciprofloxacin to ceftriaxone (i.e., from ST1 or ST2 to ST3 or ST4, Table 1). The sensitivity and specificity of the several screening tests were obtained from the peer-reviewed medical literature (14,18,19).

## Costs

Table 3 shows the cost estimates used in the model. The direct medical costs included were those associated with diagnostic testing, antimicrobial therapy for gonorrhea, and subsequent sequelae of untreated gonorrhea (15,21–23). Because the perspective of the analysis is that of the healthcare system, we did not include indirect costs, such as lost production, and intangible costs, such as pain and personal trauma.

**Table 3:** Cost estimates

Items	Costs (2001 US$)			
	Base	Range	Distribution*	Sources
Nonculture test for *Neisseria gonorrhoeae*	7	5–20	Triangular	Pers. comm.†
Culture test for *N. gonorrhoeae*	5			Pers. comm.†
Antimicrobial susceptibility tests	20	5–60	Uniform	Pers. comm.†
Weighted cost of symptomatic pelvic inflammatory disease (PID) and sequelae for untreated gonorrhea‡	3,250	3,000–3,500	Uniform	13,15,21,23
Weighted cost of asymptomatic PID and sequelae for untreated gonorrhea‡	2,250	2,000–2,500	Uniform	13,15,21,23
Outpatient case of epididymitis	229	152–277	Uniform	13,15,21,23
Inpatient case of epididymitis	3,604	2,997–4,802	Uniform	13,15,21,23
Clinic time: 5 min (routine checkup)	15	40–70	Uniform	17,24
Clinic time: 30 min (pelvic examination)	60	5–20	Uniform	17,24
Ciprofloxacin, 500 mg, oral	2	1–6	Uniform	25,26
Ceftriaxone, 125 mg, IM§	10	10–15	Uniform	25,26
Onward transmission of gonorrhea to female, per case of gonorrhea¶	60	0–300	Triangular	14,21,22,23
Onward transmission of HIV to male, per case of gonorrhea#	130	0–1,000	Triangular	20,27,28

*The probability distributions used in the Monte Carlo sensitivity analysis. Uniform distributions were constructed with the minimum and maximum of the given ranges. Triangular distributions were constructed with the minimum and maximum of the given ranges, and the base case as the "most likely" value. †Costs of culture and nonculture diagnostic tests were obtained from Dean Willis and Karla Schmitt, Florida State Department of Health. Costs of susceptibility testing were provided by Norman O'Connor, State of Hawaii Department of Health Laboratories Division, Roman Golash, Illinois Department of Public Health, and Paul Hannah, Orange County Public Health Laboratory, California. ‡Weighted using the probabilities (Table 2) of occurrence of PID (only), infertility, ectopic pregnancy, and chronic pelvic pain. Dollar values of each health outcome taken from listed sources. §IM, intramuscular injection. ¶Cost of gonorrhea transmitted to female after initial female-to-male transmission. Calculated as a weighted average cost, weighted using the probabilities of onward transmission and the probabilities of occurrence of PID (only), infertility, ectopic pregnancy and chronic pelvic pain in the female (see Table 2 for probabilities). Costs of each outcome were taken from the listed sources. #Cost of HIV in male patient after initial female-to-male transmission. Calculated as a weighted average cost, weighted by using the probabilities of onward transmission (see Table 2 for probabilities). Cost of a case of HIV is a weighted average cost, weighted by probabilities of HIV-related health outcomes, taken from listed sources.

Previous studies estimated the average clinician time associated with a full pelvic examination, including the estimated follow-up cost of scheduling a return visit for a positive test result, by direct observation of activities in a clinic patient-flow analysis (17). The direct medical costs of the time associated with a PID-related clinic visit, either an initial visit or one including a full pelvic exam, were estimated by using the MarketScan database (24). The costs of the various diagnostic tests were obtained through personal communication with health department laboratories in Hawaii, Orange County (California), and Florida (Table 3). The cost of diagnostic tests included the cost of reagents, kits, equipment, supplies, and the laboratory technician's time. For nonculture tests, since *N. gonorrhoeae* testing is routinely performed as part of a dual *N. gonorrhoeae* and *C. trachomatis* test, the incremental cost of performing the *N. gonorrhoeae* test as part of the dual test was used in the base model. However, the considerably higher cost of performing a single *N. gonorrhoeae* nonculture test by itself was incorporated and examined in the sensitivity analysis. The range of costs for antimicrobial agents reflects the prices obtained from both the private sector and public clinics (25,26). We assume directly observed therapy resulting in full treatment compliance, and any residual noncompliance is implicitly assumed as treatment failure. We estimated the average cost of both symptomatic and asymptomatic PID by summing the costs associated with each outcome multiplied by the proportion of persons who will be affected (Table 3). The principal outcomes associated with untreated infection, if symptomatic, include inpatient and outpatient treatment cost of PID and subsequent long-term chronic pelvic pain, surgery, ectopic pregnancy, and infertility. The outcomes associated with asymptomatic or silent PID are long-term sequelae only. The model also incorporated the cost of transmission of both gonorrhea and HIV to the index patient's sexual partners (20,27,28). All cost data were adjusted to 2001 US dollars, by using the medical care component of the consumer price index (29).

## Sensitivity Analysis

Univariate sensitivity analyses were conducted to examine the effect of changes in the prevalence of gonorrhea and the prevalence of ciprofloxacin-resistant *N. gonorrhoeae* on the cost per patient successfully treated. Multivariate sensitivity analyses were conducted to determine breakeven points (or threshold values) indicating input values at which any 2 strategies had the same cost per patient successfully treated. Threshold values were calculated to determine the robustness of the baseline results and the relative importance of the input variables on allowing for variation around the baseline.

To determine when a change occurs in the threshold, we simultaneously changed the values of key variables over a range of gonorrhea prevalence in women (0%–15%) and a range of prevalence of ciprofloxacin-resistant gonorrhea (0%–20%). We changed the cost ratio of ciprofloxacin to ceftriaxone from the base case of 1:5 (ciprofloxacin = $2/dose; ceftriaxone = $10/dose; Table 3) to both 1:2 and 1:7.5. Simultaneously, we changed the cost ratio of culture tests to nonculture tests from 1:1.4 (culture tests = $5, nonculture tests = $5, Table 3) to both 1:1 and 1:3. We then simultaneously altered the specificity and sensitivity of the 2 tests. Finally, we conducted a Monte Carlo simulation,^2^ in which we simultaneously altered all the input variables by using predefined probability distributions to examine whether they had significant consequences on model results.

## Results

### Base Case Analysis

All 4 strategies ensured that PID did not develop in >99% of all treated patients, regardless of the assumed prevalence of gonorrhea (Table 4). This finding means that the costs per patient treated are almost the same as the cost per patient successfully treated (i.e., costs per patient with no PID or sequelae) and that relative costs are central in determining cost-effectiveness. However, the high cost-effectiveness ratios (CERs, which estimate the additional cost per additional case of PID averted on comparing a strategy with the baseline or the next-most-effective strategy in average and incremental cost-effectiveness analysis, respectively) generated in cases in which alternative strategies are similar in effectiveness do not offer an intuitive decision-making tool for choosing an optimal strategy. Instead, a cost-minimization approach, which selects as optimal a strategy that minimizes cost per case successfully prevented (i.e., least costly in achieving the same level of effectiveness), provides a more practical and intuitive decision-making tool. Detailed results from incremental cost-effectiveness analyses are contained in Appendix 2 for those programs that choose to consider the additional CERs in making decision on budgetary allocations.

**Table 4:** Cost per case treated and percentage of treated cases without PID* on varying prevalence of gonorrhea and ciprofloxacin resistance (base-case values†)

Prevalence (%) gonorrhea‡	Strategy§	0.1%		2%		10%	
		$/case treated¶	% cases with no PID¶	$/case treated	% cases with no PID	$/case treated	% cases with no PID
1	ST1	26.00	99.92	26.03	99.92	26.17	99.92
	ST2	32.76	99.93	32.85	99.93	33.20	99.92
	ST3	26.21	99.92	26.21	99.92	26.21	99.92
	ST4	34.07	99.93	34.07	99.93	34.07	99.93
5	ST1	42.04	99.61	42.20	99.60	42.89	99.60
	ST2	45.70	99.65	46.11	99.64	47.87	99.61
	ST3	41.92	99.61	41.92	99.61	41.92	99.61
	ST4	47.12	99.65	47.12	99.65	47.12	99.65
10	ST1	62.09	99.21	62.41	99.21	63.79	99.18
	ST2	61.86	99.30	62.70	99.29	66.21	99.22
	ST3	61.55	99.21	61.55	99.21	61.55	99.21
	ST4	63.42	99.31	63.42	99.31	63.42	99.31

*PID, pelvic inflammatory disease, which can cause sequelae such as chronic pelvic pain, infertility, and ectopic pregnancy. †Baseline values given in Tables 2 and 3. ‡When gonorrhea prevalence is 1% and prevalence of ciprofloxacin-resistant *Neisseria gonorrhoeae* is 0.1%, PID would not develop in 98.4% of patients treated. In the absence of any treatment, PID would not develop in 74% (range 60%–90%) of gonorrhea-infected women. §Strategies modeled were ST1: ciprofloxacin + culture-based tests + ciprofloxacin-susceptibility tests; ST2: ciprofloxacin + nonculture-based tests; ST3: ceftriaxone + culture-based tests + ceftriaxone-susceptibility tests; ST4: ceftriaxone + nonculture-based tests. See Table 1 and text for further details. ¶Cost per patient treated and percentage of patients treated refer to all women who come to the public health clinic and undergo therapy as per 1 of the 4 strategies, regardless of actual infection.

When the prevalence of gonorrhea is ≤5%, the 2 strategies based on culture and susceptibility testing (ST1 and ST3) are cheaper than the other 2 strategies (ST2 and ST4). For any strategy, increasing the prevalence of gonorrhea from 1% to 10% more than doubled the cost per patient treated. This doubling is primarily due to the increase in the proportion of patients who face additional costs for testing, treatment, or both.

**Figure 1:**
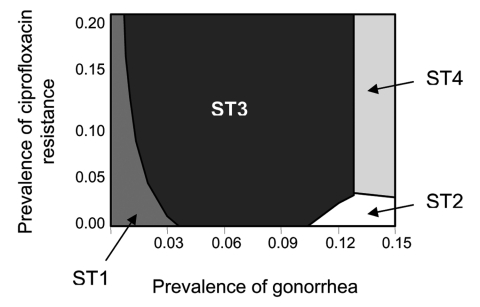
Lowest cost per patient successfully treated on varying prevalence of gonorrhea and prevalence of ciprofloxacin-resistant *Neisseria gonorrhoeae*. Notes: strategy depicted is optimal (lowest cost per patient successfully treated) for given combinations of prevalence of gonorrhea and prevalence of ciprofloxacin-resistant *N. gonorrhoeae*. Since the alternative strategies are similar in effectiveness, cost-effectiveness analysis does not offer a practical decision-making tool. Instead, cost minimization, which selects as optimal a strategy that costs least while achieving the same level of effectiveness (i.e., per case of successful treatment), serves as a more practical and intuitive tool kit for decision making. Case-patients refer to all women who attend a public health clinic and undergo therapy as per 1 of the 4 strategies, regardless of actual infection. The strategies modeled were ST1: ciprofloxacin + culture-based tests + ciprofloxacin-susceptibility tests; ST2: ciprofloxacin + nonculture-based tests; ST3: ceftriaxone + culture-based tests + ceftriaxone-susceptibility tests; ST4: ceftriaxone + nonculture-based tests (see Table 1 and text for further details). Values for input variables other than prevalence of gonorrhea and prevalence of ciprofloxacin-resistant *N. gonorrhoeae* are the base case values given in Tables 2 and 3.

Results from varying the prevalence of gonorrhea and the prevalence of ciprofloxacin resistance simultaneously are shown in Figure 1. If the prevalence of gonorrhea is <1%, ST1 has the lowest cost per patient successfully treated even if prevalence of ciprofloxacin resistance is as high as 20%. Even when prevalence of gonorrhea approaches 3%, ST1 is the optimal strategy if prevalence of ciprofloxacin resistance is <4%. Strategy 3 (ceftriaxone + culture-based testing) is frequently the most optimal strategy when prevalence of gonorrhea is 3%–12%. With a few exceptions, the 2 strategies that use nonculture-based tests become most optimal only when the prevalence of gonorrhea is >13%. Finally, if ciprofloxacin-resistance levels are ≥3% and gonorrhea prevalence is >13%, a switch to ceftriaxone (ST4) is recommended. Overall, the base-case analysis indicates that culture-based strategies are optimal (lowest cost per patient successfully treated) at lower levels of gonorrhea prevalence, while nonculture-based strategies become optimal as gonorrhea prevalence increases.

### Sensitivity Analysis

**Figure 2:**
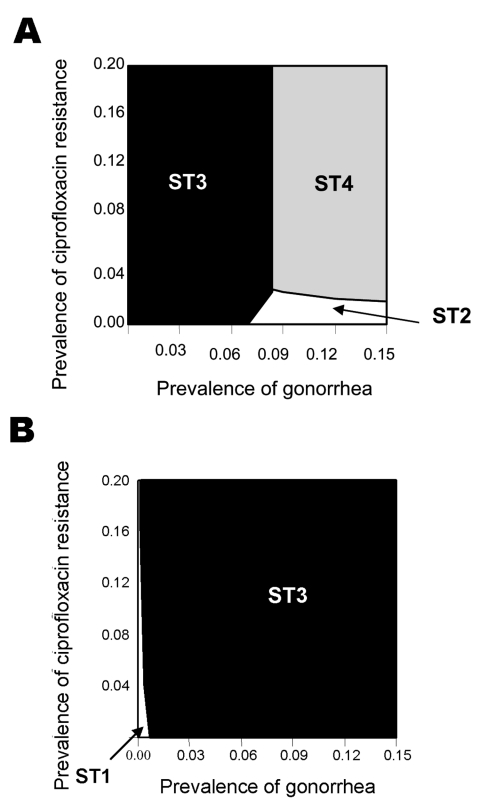
Lowest cost per patient successfully treated on varying relative costs of drugs and tests. A) Cost of culture = $5; cost of nonculture = $5; cost of ciprofloxacin= $5; cost of ceftriaxone = $10. B) Cost of culture = $5; cost of nonculture = $15; cost of ciprofloxacin = $2; cost of ceftriaxone=$15. For notes, see Figure 1 legend.

The model was found to be sensitive to changes in several estimates, including the relative cost of antimicrobial agents and diagnostic tests. For example, if the ratio of cost of ciprofloxacin to cost of ceftriaxone is changed from 1:5 (base case, Figure 1) to 1:2 (Figure 2A) and the costs of tests become equal (Figure 2A), the two strategies that include non-culture tests (ST2 and ST4) are optimal for greater combinations of gonorrhea prevalence and ciprofloxacin-resistance prevalence than in the base case. However, if the ratio of the cost of culture tests to non-culture tests is changed from 1:1 (Figure 2A) to 1:3 (Figure 2B), then the two strategies that include culture tests (ST1 and ST3) become optimal for all combinations of gonorrhea prevalence and ciprofloxacin-resistance prevalence.

**Figure 3:**
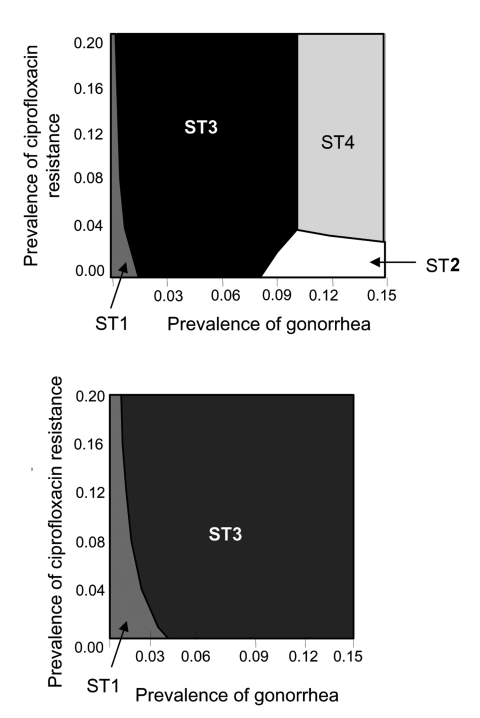
Lowest cost per patient successfully treated on varying sensitivity and specificity of culture- and nonculture-based tests. A) Culture: sensitivity = 75%, specificity = 95%; nonculture: sensitivity = 85%, specificity = 95%. For notes, see Figure 1 legend. B) Culture: sensitivity = 95%, specificity = 97%; nonculture: sensitivity = 85%, specificity = 95%.

Regardless of the relative difference in sensitivity and specificity of the 2 types of tests, strategies containing culture-based tests (ST1 or ST3) are optimal if prevalence of gonorrhea is <6% (Figure 3A). However, when both the sensitivity and specificity of the culture-based tests are set at the minimum values, and the nonculture-based tests are at maximum values, the optimal diagnostic choice switches from culture-based (ST1 or ST3) to nonculture-based tests (ST2 or ST4), if prevalence of gonorrhea is ≥8% (Figures 3A). However, when the sensitivity and specificity of culture-based tests are set at their maximum value, and the nonculture-based tests are at their minimum value, the 2 strategies that contain culture-based tests are optimal for all combinations of gonorrhea prevalence and ciprofloxacin-resistance prevalence (Figure 3B).

From the Monte Carlo analysis, for 3 of the combinations of gonorrhea prevalence and ciprofloxacin-resistance prevalence, strategy 1 has the lowest mean cost per patient treated (Table 5). Only when gonorrhea prevalence is 2% and ciprofloxacin-resistance prevalence is 10% does strategy 3 have a lower mean cost per patient treated (Table 5). Note that, for any given combination of gonorrhea prevalence and ciprofloxacin-resistance prevalence, considerable overlap exists among the confidence intervals around the means of the cost per patient treated (Table 5).

**Table 5:** Monte Carlo simulation* results: mean cost per patient treated and percentage of patients without PID† >(5th percentile, 95th percentile)

Prevalence (%) gonorrhea	Strategy‡	Prevalence of ciprofloxacin resistance = 0.1%		Prevalence of ciprofloxacin resistance = 2%	
		$/patient treated	% patients without PID	$/patient treated	% patients without PID
1	ST1	27.34 (21.45, 33.23)	99.92 (99.84, 99.96)	27.44 (21.72, 33.64)	99.92 (99.84, 99.96)
	ST2	39.78 (30.45, 50.19)	99.92 (99.83, 99.96)	39.78 (30.57, 50.95)	99.92 (99.84, 99.96)
	ST3	28.94 (22.92, 35.08)	99.92 (99.84, 99.96)	28.99 (23.25, 35.32)	99.92 (99.84, 99.96)
	ST4	42.00 (31.38, 53.08)	99.93 (99.84, 99.96)	41.99 (32.23, 53.83)	99.92 (99.84, 99.96)
10	ST1	68.73 (46.22, 99.01)	99.18 (98.41, 99.59)	71.65 (47.04, 102.58)	99.16 (98.38, 99.61)
	ST2	77.34 (53.53, 106.86)	99.23 (98.43, 99.63)	77.29 (54.83, 110.64)	99.19 (98.44, 99.61)
	ST3	70.37 (47.06, 98.72)	99.18 (98.41, 99.60)	70.33 (46.85, 101.75)	99.17 (98.39, 99.61)
	ST4	79.69 (55.78, 109.48)	99.23 (98.44, 99.80)	79.68 (55.90, 110.87)	99.21 (98.48, 99.63)

*Monte Carlo simulation involves specifying a probability distribution of values for model inputs (see Tables 2 and 3 for distributions used). A computer algorithm ran the model for 10,000 iterations. During each iteration, the computer algorithm selects input values from the probability distributions and calculates the output (e.g., cost per patient successfully treated). After the final run, the model provides results such as the mean, median, and 5th and 95th percentiles for each specified output. †PID, pelvic inflammatory disease, which can cause sequelae such as chronic pelvic pain, infertility, and ectopic pregnancy. ‡The strategies modeled were ST1: ciprofloxacin + culture-based tests + ciprofloxacin-susceptibility tests; ST2: ciprofloxacin + nonculture-based tests; ST3: ceftriaxone + culture-based tests + ceftriaxone-susceptibility tests; ST4: ceftriaxone + nonculture-based tests. See Table 1 and text for further details.

## Discussion

The absence of any recommended, evidence-based method that can be used to identify the most cost-effective gonorrhea treatment strategy has resulted in ad hoc decision making regarding when to change drug therapy because of antimicrobial resistance. For example, the threshold for changing drug therapy for gonorrhea treatment has often been when prevalence of gonococcal strains resistant to a given antimicrobial agent reaches 5% (30–32). The model indicates that using a single variable to define the breakpoint is inefficient. For example, if gonorrhea prevalence is <1%, our results show that ciprofloxacin would be most optimal even if ciprofloxacin resistance were as high as 20%. The decision regarding when to change from 1 drug to another on the basis of the prevalence of gonorrhea and the prevalence of ciprofloxacin resistance is summarized in a tool kit contained in Appendix 3.

While we cannot cover every situation and setting, our results clearly illustrate that a single, generic policy regarding when to switch drug treatments (for reasons of antimicrobial resistance) is not necessarily optimal from an economic perspective. However, the sensitivity analyses demonstrate that our model has wide applicability and can, by varying input data, provide answers across a wide range of settings. The current model can readily be adopted to produce a practical and interactive tool kit that would allow for variation across a wide range of input values.

Our analyses identified 2 other important points. First, since all the strategies were similar in terms of effectiveness (i.e., percentage of patients successfully treated), relative costs will be important in determining the most cost-effective strategy. Second, the large variability in key variables (i.e., prevalence of gonorrhea, prevalence of ciprofloxacin-resistance, relative costs of drugs and diagnostic tests) across geographic locations and clinical settings makes it unlikely that the same single strategy will be the most cost-effective strategy across all these settings.

The sensitivity of the results to relative costs of diagnostic tests is of concern because the current practice of providing higher reimbursement rates (compared to actual cost) for nonculture serves as a subsidy for nonculture tests. We also did not value all the benefits associated with culture-based tests, specifically the additional knowledge obtained regarding antimicrobial susceptibility. In interpreting the model results, the inadequacies of not accounting for the full benefits of culture should be acknowledged. Likewise, nonculture-based testing, which does not necessarily require a pelvic examination, may confer both cost advantages and higher patient acceptability (e.g., noninvasive methods for testing may be preferred by some women).

Further, in practice, selection of diagnostic test is often driven by priorities of testing for chlamydia, rather than gonorrhea testing alone. We did not consider all the costs and benefits associated with diagnosis and treatment of both *N. gonorrhoeae* and *C. trachomatis*. In addition, our results apply specifically to adult women and cannot be generalized for men.

With regard to antimicrobial drug selection, Monte Carlo simulations, which were based on assumed distributions and not actual data, show considerable overlap in costs and effectiveness across the 2 antimicrobial choices. Accordingly, caution should be exercised in recommending 1 drug over another, unless the results are backed with more certain and site-specific data on key variables for a given location.

A limitation that should prevent overemphasizing the sensitivity of the results to the relative cost of the drugs is the assumption of 100% treatment failure with ciprofloxacin resistance, which may overestimate the cost of ciprofloxacin resistance and incomplete patient recalls. Our model also assumes that resistance (or other treatment failures) to ceftriaxone is zero (as per the latest surveillance reports), although the model is designed to allow one to relax the assumption and vary the prevalence of ceftriaxone resistance. If one were to assume <100% treatment failure with ciprofloxacin or assume existence of some treatment failure to ceftriaxone including resistance, using ciprofloxacin (ST1 and ST2) would be most cost-effective for even larger ranges of gonorrhea prevalence and ciprofloxacin-resistance prevalence.

If a single strategy has a greater probability of contributing to resistance (because of inappropriate antimicrobial use), measuring the additional cost of increased resistance is beyond the scope of this model. A model limitation also arises from not including a valuation for reserving a class of antimicrobial agents for future use. Our model contains the implicit assumption that when ceftriaxone-resistant gonorrhea becomes problematic, an equally effective and affordable antimicrobial agent will be available to replace ceftriaxone. If the future costs of prematurely depriving physicians and patients of ceftriaxone were included, strategy 1 would become the dominant strategy in Figure 1. Any method used to recommend systemwide switching of drug therapies because of antimicrobial resistance should take into account that considerable value exists in keeping in reserve an already existing antimicrobial agent for as long as economically feasible.

The overall conclusion from our model is that decisions regarding changes in drug therapies used for gonorrhea treatment require several types of data. Both prevalence of gonorrhea and prevalence of ciprofloxacin-resistant gonococcal strains must be considered. Since prevalence data are dynamic and population-specific, ongoing collection of such data is necessary to allow informed decision making to take place.

## Appendix 1

### Decision Trees

Following are links to schematic diagrams showing the decision trees used to produce the results presented in this article.

**Figure A1:**
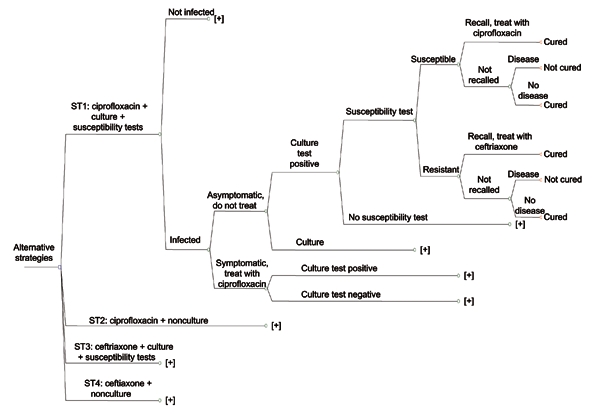
The 4 strategies; + denotes "truncated" branch; GC, gonorrhea.

Figure A1. The 4 strategies; + denotes "truncated" branch; GC, gonorrhea.

**Figure A2:**
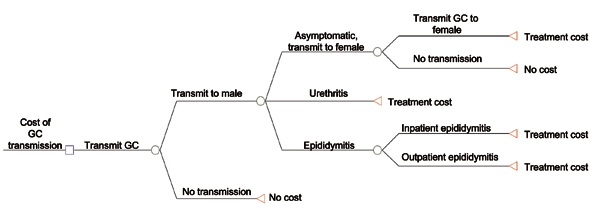
Cost of gonorrhea transmission.

Figure A2. Cost of gonorrhea transmission.

**Figure A3:**
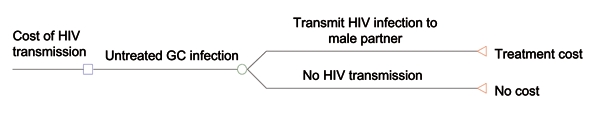
Cost of HIV transmission. GC, gonorrhea.

Figure A3. Cost of HIV transmission. GC, gonorrhea.

**Figure A4:**
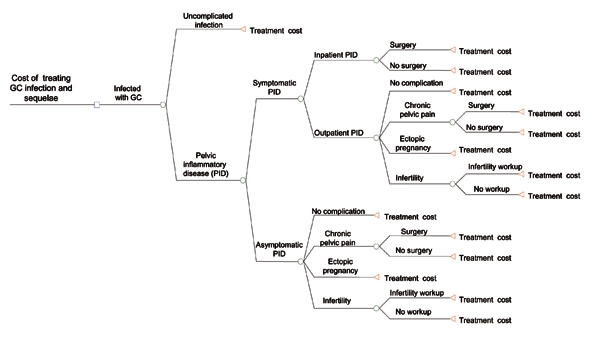
Weighted cost of treating gonorrhea infection and sequelae in 2001 US dollars.

Figure A4. Weighted cost of treating gonorrhea infection and sequelae in 2001 US dollars.

## Appendix 2

### Average and Incremental Cost-effectiveness Analysis

Average and incremental cost-effectiveness analyses conducted for a hypothetical cohort of 1 million women treated with each of the 4 alternative strategies is presented in the Table A1, below. Average cost-effectiveness was estimated as the cost per case successfully treated with a given strategy compared to the baseline strategy. Incremental cost-effectiveness ratio was estimated as the additional cost per additional case of pelvic inflammatory disease (PID) averted for a strategy compared to the next less effective strategy.

Cost per case prevented varies depending on prevalence of gonorrhea (PR_GC_) and prevalence of ciprofloxacin resistance (PR_CIPRO_). Using base-case estimates], and assuming that PR_GC_ is 1% and PR_CIPRO_ is 0.1%, the resulting cost-effectiveness ratios (CERs) indicate that ST3 (ceftriaxone + culture) is strongly dominated by ST1 (ciprofloxacin + culture). The costs per case of PID prevented compared to the baseline (ST1) for ST2 (ciprofloxacin + nonculture) and ST4 (ceftriaxone + nonculture) are $356,087 and $366,344, respectively. Incremental cost-effectiveness analysis indicates that ST2 compared to ST1 costs an additional $73,478 per case prevented, and ST4 compared to ST2 costs an additional $8,070,000 per case prevented. However, if PR_GC_ is 10%, even with PR_CIPRO_ at 0.1%, ST1 and ST3 are strongly dominated by ST2. Thus, nonculture-based strategies (ST2 and ST4) are more cost-effective than culture-based strategies (ST1 and ST3), and the cost per case of PID prevented by ST4 compared to ST2 is $173,000.

## Appendix 3

### Tool Kit for Decision Making across Different Scenarios

Average and incremental cost-effectiveness analyses conducted for a hypothetical cohort of 1 million women treated with each of the 4 alternative strategies is presented in the Table A2, below. Average cost-effectiveness was estimated as the cost per case successfully treated with a given strategy compared to the baseline strategy. Incremental cost-effectiveness ratio was estimated as the additional cost per additional case of pelvic inflammatory disease (PID) averted for a strategy compared to the next less effective strategy.

## Footnotes

1 In 2000, only 18% of gonorrhea tests performed by public health laboratories in the United States were culture-based tests.

2 Monte Carlo simulation involves specifying a probability distribution of values for model inputs. A computer algorithm then runs the model for several iterations. During each iteration, the computer algorithm selects input values from the probability distributions, and calculates the output (e.g., cost per patient successfully treated). After the final run, the model provides results such as the mean, median, and 5th and 95th percentiles for each specified output.

## Appendix

**Table A1:** Average and incremental cost-effectiveness analysis* for a cohort of 1 million women (prevalence of ciprofloxacin resistance = 0.1%)

Alternative strategies (from least to most effective)	Expected number of cases of PID†	Total cost (intervention + sequelae) (US $1,000s)	Incremental cost	Average cost-effectiveness‡ ratio	Incremental cost-effectiveness ratio§
*Neisseria gonorrhoeae* prevalence = 0.01					
ST1: ciprofloxacin + culture	787	$26,000	__	Baseline	Baseline
ST3: ceftriaxone + culture	787	$26,210	$210,000	(Strongly dominated)¶	(Strongly dominated)¶
ST2: ciprofloxacin + nonculture	695	$32,760	$6,760,000	$356,087	$73,478
ST4: ceftriaxone + nonculture	694	$34,070	$8,070,000	$366,344	$8,070,000
*N. gonorrhoeae* prevalence = 0.10					
ST1: ciprofloxacin+ culture	7,874	$62,090	__	(Strongly dominated)#	(Strongly dominated)#
ST3: ceftriaxone + culture	7,871	$62,090	__	(Strongly dominated)#	(Strongly dominated)#
ST2: ciprofloxacin + nonculture	6,953	$61,860	__	Baseline	Baseline
ST4: ceftriaxone + nonculture	6,941	$63,420	$1,560,000	$7,046,000	$173,000

*Applies baseline values to all variables, other than prevalence of *N. gonorrhoeae.* †PID (pelvic inflammatory disease) includes cases of both symptomatic and asymptomatic PID and sequelae. If gonorrhea prevalence is 1%, 1,600 cases of PID would result in the absence of any intervention. If the prevalence of gonorrhea is 10%, the number of PID cases would be 16,000. However, "do nothing" is not a feasible strategy for a clinic as it has already committed to treatment of sexually transmitted diseases. ‡Cost-effectiveness ratios are expressed as cost (in thousands of dollars) per additional case of PID prevented compared to the baseline strategy. § Incremental cost-effectiveness ratios are expressed as cost (in thousands of dollars) per additional case of PID prevented compared to the least expensive strategy listed in the preceding row. ¶A strongly dominated strategy is one that is more expensive than an equally or a more effective strategy. For example, ST3 is strongly dominated by ST1 as it is equally effective but more expensive than ST1. #Both ST1 and ST3 are strongly dominated by ST2 as they are both strategies that are less effective but more expensive than ST2.

**Table A2:** Tool kit for decision-making

Prevalence of gonorrhea, %	Prevalence of ciprofloxacin resistance, %	Optimal strategy*,†,‡
0–1	0–20	ST1: ciprofloxacin + culture
2–3	0–5	ST1
2–3	>5	ST3: ceftriaxone + culture
3–10	0–20	ST3
10–13	0–3	ST2: ciprofloxacin + nonculture
10–13	>3	ST3
13–15	0–3	ST2
13–15	>3	ST4: ceftriaxone + nonculture

*Optimal strategy is the one that yields the lowest cost per case successfully treated for given combinations of prevalence of gonorrhea and prevalence of ciprofloxacin-resistant *Neisseria gonorrhoeae*. †Since the alternative strategies are similar in effectiveness, cost-effectiveness analysis may not offer a practical decision-making tool. Instead, cost minimization which selects as optimal a strategy that costs the least while achieving the same level of effectiveness (i.e., per case of successful treatment) may serve as a more practical and intuitive toolkit for decision-making. ‡The above table shows the choice of an optimal strategy (lowest cost per case successfully treated) on varying the prevalence of gonorrhea and prevalence of ciprofloxacin resistance across several geographic settings. All other variables are assumed to have baseline values.
